# Distal Effect of Amino Acid Substitutions in CYP2C9 Polymorphic Variants Causes Differences in Interatomic Interactions against *(S)*-Warfarin

**DOI:** 10.1371/journal.pone.0074053

**Published:** 2013-09-02

**Authors:** Panida Lertkiatmongkol, Anunchai Assawamakin, George White, Gaurav Chopra, Pornpimol Rongnoparut, Ram Samudrala, Sissades Tongsima

**Affiliations:** 1 Department of Biochemistry, Faculty of Science, Mahidol University, Bangkok, Thailand; 2 Genomics Institute, National Center for Genetic Engineering and Biotechnology, Pathumtani, Thailand; 3 Department of Microbiology, University of Washington, Seattle, Washington, United States of America; 4 Department of Pharmacology, Faculty of Pharmacy, Mahidol University, Bangkok, Thailand; 5 Diabetes Center, University of California San Francisco, San Francisco, California, United States of America; Wake Forest University, United States of America

## Abstract

Cytochrome P450 2C9 (CYP2C9) is crucial in excretion of commonly prescribed drugs. However, changes in metabolic activity caused by CYP2C9 polymorphisms inevitably result in adverse drug effects. CYP2C9*2 and *3 are prevalent in Caucasian populations whereas CYP2C9*13 is remarkable in Asian populations. Single amino acid substitutions caused by these mutations are located outside catalytic cavity but affect kinetic activities of mutants compared to wild-type enzyme. To relate distal effects of these mutations and defective drug metabolisms, simulations of CYP2C9 binding to anti-coagulant (*S*)-warfarin were performed as a system model. Representative (*S*)-warfarin-bound forms of wild-type and mutants were sorted and assessed through knowledge-based scoring function. Interatomic interactions towards (*S*)-warfarin were predicted to be less favorable in mutant structures in correlation with larger distance between hydroxylation site of (*S*)-warfarin and reactive oxyferryl heme than wild-type structure. Using computational approach could delineate complication of CYP polymorphism in management of drug therapy.

## Introduction

Cytochrome P450 (CYP) is a superfamily of oxygen-activating enzymes, which metabolize a broad range of compounds and transform hydrophobic drugs into soluble forms. CYP polymorphism can affect drug metabolism and result in adverse drug reaction (ADR). Therefore, pharmacogenetics should be considered for the maximal efficacy of the treatment.

CYP2C9 contributes to metabolism of over 100 prescribed drugs such as (*S*)-warfarin, losartan and diclofenac [1]. Single nucleotide polymorphisms (SNPs) of CYP2C9 with functional abnormalities have been reported [2]. Metabolic activity of most polymorphic variants is decreased compared to the wild-type CYP2C9 [3], leading to ADR. Defective effects of SNPs have been reported in metabolism of approximately 20 drugs [4], [5] while over 80 drugs have not yet been characterized. Metabolism of (*S*)-warfarin is dramatically affected by SNPs due to remarkable narrow therapeutic index [6]. Thus, (*S*)-warfarin drug therapy management is varied among individuals because of differences in metabolic rates of CYP2C9 polymorphic isoforms.

The predominant alleles found in Caucasians, CYP2C9*2 and CYP2C9*3 [7], [8], cause amino acid substitutions R144C and I359L, respectively, that are not in the active site of the enzyme but decreased the maximal rate (*V*_max_) of (*S*)-warfarin metabolism compared with wild-type CYP2C9 [9]. However, catalytic activity implemented by Michaelis-Menten constant (*K*_m_) of R144C variant was similar to the wild-type enzyme whereas I359L variant led to 3-fold reduction in *K*_m_ value [9]. The mean oral clearance of (*S*)-warfarin was observed *in vivo* with a reduction of 56% for CYP2C9*2/*2 and 89% for CYP2C9*3/*3 individuals [10], corresponding to 36% and 78.1% dose reduction compared to CYP2C9*1/*1 [11]. CYP2C9*3/*13 also required 70% decrease to achieve effective treatment [12]. Impaired metabolisms of lornoxicam and losartan were identified in CYP2C9*13 affected individuals [13], [14]. Despite the high incidence of CYP2C9*13 allele in Asian population [15]–[17], the effect of CYP2C9*13 on (*S*)-warfarin metabolism is not clearly elucidated.

X-ray crystal structures of CYPs reveal 12 helices (A-L) and 4 sheets (β1–4) shared from prokaryotic to eukaryotic CYPs [18]. Variable regions interacting with substrates were described as substrate recognition sites (SRSs) [19], spanning the active-site cavity and substrate access channels. Unexpectedly, most mutations found in defective CYP polymorphisms are located outside SRSs [3]. To investigate distal effects of mutations, molecular dynamics (MD) simulations demonstrated that R144C, I359L, and D360E arising from CYP2C9*2, *3, and *5, respectively, were not located within the substrate-binding site and/or active site but resulted in an increased fluctuation of residues holding substrates and changed enzymatic activity [20], [21]. Furthermore, reduction in substrate access channel size of L90P, R144C and I359L could hinder entry of substrate into active site [21], [22]. However, how the substituted residues cause structural changes remains unclear.

To explain functional abnormality of polymorphic CYP2C9, we performed MD simulations to generate (*S*)-warfarin-bound structural ensembles of each variant. Knowledge-based discriminatory function was then utilized to evaluate interatomic interactions between (*S*)-warfarin and binding cavity in each of the generated structures to distinguish native-like bound structures from non-native structures. Dominant structural motions of each CYP2C9 variant were determined by principle component analysis (PCA) to illustrate structural optimization. Furthermore, structural effect of CYP2C9*13 on (*S*)-warfarin metabolism was predicted. The proposed structural analysis method revealed distal effects of mutated residues that could alter atomic interactions between residues inside binding pocket and (*S*)-warfarin. Consequently, metabolic activity of these CYP2C9 variants could be reduced whereas substrate selectivity might not be changed. The proposed method can be utilized for investigation of variable drug response among poor drug-metabolizers.

### Computational Methods

All computational studies were performed on AMD64-based cluster running Rocks (Version 5.1) with Centos 5.3 Linux operating system. Molecular visualization was performed on PyMOL 0.93 (Schrödinger, LLC).

#### Construction of WT, R144C, I359L, and L90P structures

Ligand-bound crystal structure of CYP2C9 (PDB: 1OG5 [23]) was used as a starting structure for construction of substrate-free forms of wild-type (WT) and mutants (R144C, I359L, and L90P). Seven amino acid substitutions of K206E, I215V, C216Y, S220P, P221A, I223L and I224L were introduced in 1OG5 in order to enhance crystallization. Thus, these mutations were modified back to WT sequence using SCWRL 3.0 [24], and the wild-type structure was subsequently mutated to R144C, I359L, and L90P for CYP2C9*2, *3 and *13 mutations.

#### Re-docking of (S)-warfarin in WT

In (*S*)-warfarin-bound complex (PDB: 1OG5), (*S*)-warfarin is located approximately 10 Å away from the heme center and is not in a catalytically productive orientation for monooxygenation reaction [25]. Therefore, three-dimensional structure of (*S*)-warfarin obtained from 1OG5 was applied in re-docking for a putative catalytic conformation of (*S*)-warfarin in active-site cavity of WT that could yield a major metabolite 7-hydroxywarfarin [26], using AutoDock 4.0 [27]. Partial charges of (*S*)-warfarin and protein were generated by AutoDockTools [28] using Gasteiger method. Partial charges of the heme group in oxyferryl state were assigned as previously described [29]. A cubic grid containment having 50×80×60 grid points per side with spacing of 0.375 Å was constructed to overlay SRSs of CYP2C previously proposed [19]. Affinity maps of grids were calculated using AutoGrid program. AutoDock 4.0 was employed to dock (*S*)-warfarin into WT active-site cavity using Larmarckian genetic algorithm, consisting of 200 runs and 270,000 generations, with maximum number of energy evaluation set to 2.5 × 10^6^. Resulting docked conformations within 2.0 Å root mean square deviation (RMSD) tolerance were clustered and analyzed using AutoDockTools. Conformation of (*S*)-warfarin occupying in vicinity to heme center of WT with the lowest estimated free energy of binding obtained from semi-empirical free energy force field in AutoDock4.0 [30] was selected as the bound form for all following work.

#### Molecular Dynamics (MD) simulations of WT, R144C, I359L, and L90P

Binding pockets of CYPs are remarkably flexible to accommodate ligands [31]. To obtain a set of probable (*S*)-warfarin-bound complex conformations of CYP2C9 variants in this study, MD simulations were performed using the docked conformation of (*S*)-warfarin positioned adjacent to the heme center. The all-atom AMBER force field “ff03” was used to represent the protein system whereas partial charges of oxyferryl heme were assigned as in the re-docking. All of the three-dimensional models were solvated in TIP3P water molecules with minimum distance of 12 Å between the outermost protein atoms and the water box. All systems were neutralized with sodium ions. SANDER program of AMBER10 [32] was employed in following computations. Initial structures were energy minimized for 2,000 steps of steepest descent and 3,000 steps of conjugate gradient. Simulations were performed at constant pressure and temperature of 300 K. SHAKE algorithm was applied to all bonds containing hydrogen atoms and a time step of 2 fs was used. Simulations were restrained for 20 ps and unrestrained for 25 ns. Two replica simulations were performed for each variant.

#### Trajectory analyses

To evaluate equilibration during simulations of CYP2C9 variants, RMSD of the main chain C, O, and N atoms were calculated using PTRAJ module of AMBER10. To detect dominant motion of residues in each structure, principle component analysis (PCA) was performed using PTRAJ, Perl, R, and VMD scripts developed in-house. Protein coordinates were extracted to PDB format at 10 ps intervals from the last 4 ns of each simulation. To eliminate global translational and rotational motions during simulations, trajectory coordinates were fit to the initial crystal structure. Covariance matrix of three-dimensional coordinates of C-alpha atoms was calculated using Perl scripts, and matrix was diagonalized using singular value decomposition routine in R to obtain eigenvectors, or principle components, and eigenvalues, or principle amplitudes of the matrix. The first eigenvector is illustrated as arrows overlaid on the ‘porcupine plots’ of crystal structure, created by VMD program. The starting point of each arrow in the plots represents crystal structure coordinates of corresponding C-alpha atom whereas the ending point is crystal structure coordinates plus corresponding component of eigenvector multiplied by an arbitrary scale factor that was selected by eye to aid in visualization of the eigenvectors. Same arbitrary scale factor was applied to all C-alpha atoms in each figure. Consequently, relative length of each arrow is not affected by the scale factor.

**Figure 1:**
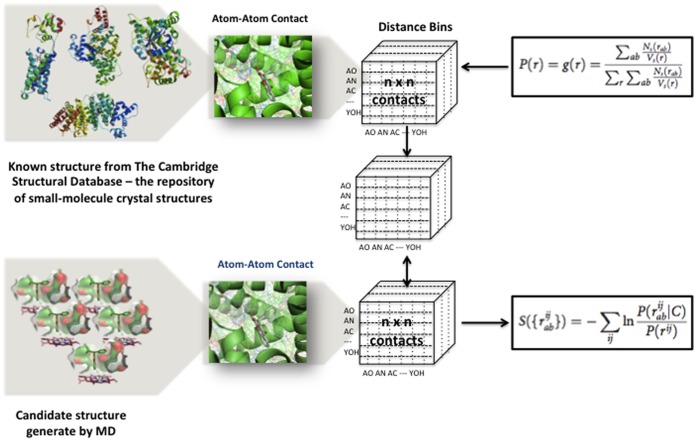
Discrimination of near native-like (*S*)-warfarin-bound complex from stable conformations obtained from MD simulations. A set of conformations of (*S*)-warfarin-bound complex were generated by MD simulations. Near native-like complex of each variant was sorted from the set of conformations by knowledge-based distribution functions that consider interatomic contacts at varied distances. The distance bins are derived from interatomic contacts between small molecules and proteins deposited in Cambridge Structural Database (CSD).

To assess (*S*)-warfarin-bound forms from the last 4 ns MD simulations of each variant, knowledge-based scoring function so called “radial distribution function with mean reference state” (r⋅m⋅r) was used to distinguish native and near native complexes from incorrect or unphysical ones [33]. For a given complex, r⋅m⋅r score is log of the sum of the values of a distribution function evaluated for each intermolecular atomic distance between enzyme and substrate, where distribution function is constructed from distributions of intermolecular atomic distances observed in the Cambridge Structural Database (CSD) [34]. In this application, interatomic interactions were calculated between (*S*)-warfarin against residues comprising binding pocket and oxyferryl heme of the enzyme. Snapshot with the lowest total score is said to exhibit the nearest native-like biomolecular interaction between substrate and enzyme that could represent (*S*)-warfarin-bound complex of each variant from the conformation set of the last 4 ns trajectory as depicted in Figure 1.

## Results

### Re-docking of (S)-warfarin

(*S*)-warfarin was re-docked into binding pocket of WT as the orientation of (*S*)-warfarin in the X-ray crystal structure of CYP2C9 (PDB ID: 1OG5) might not result in catalytic activity of CYP2C9. Putative docked conformation was subsequently determined using AutoDock4.0 [30] for which the distance between (*S*)-warfarin atoms and molecular oxygen ligated on the heme iron is favorable for monoxygenation reaction. This docked structure with (*S*)-warfarin in complex with CYP2C9 is similar to that of flurbiprofen-bound CYP2C9 crystal structure (PDB ID: 1R9O [35]). Seven-hydroxylation site of (*S*)-warfarin of the selected docking conformation poses 3.12 Å toward oxyferryl heme, with −6.26 kcal/mol of binding energy as estimated by AutoDock. The same orientation of re-docked (*S*)-warfarin in the binding pocket of WT was also placed in binding pocket of mutated structures of R144C, I359L, and L90P. Interactions between (*S*)-warfarin and CYP2C9 variants were further optimized by MD simulations.

### Structural Analyses

**Figure 2:**
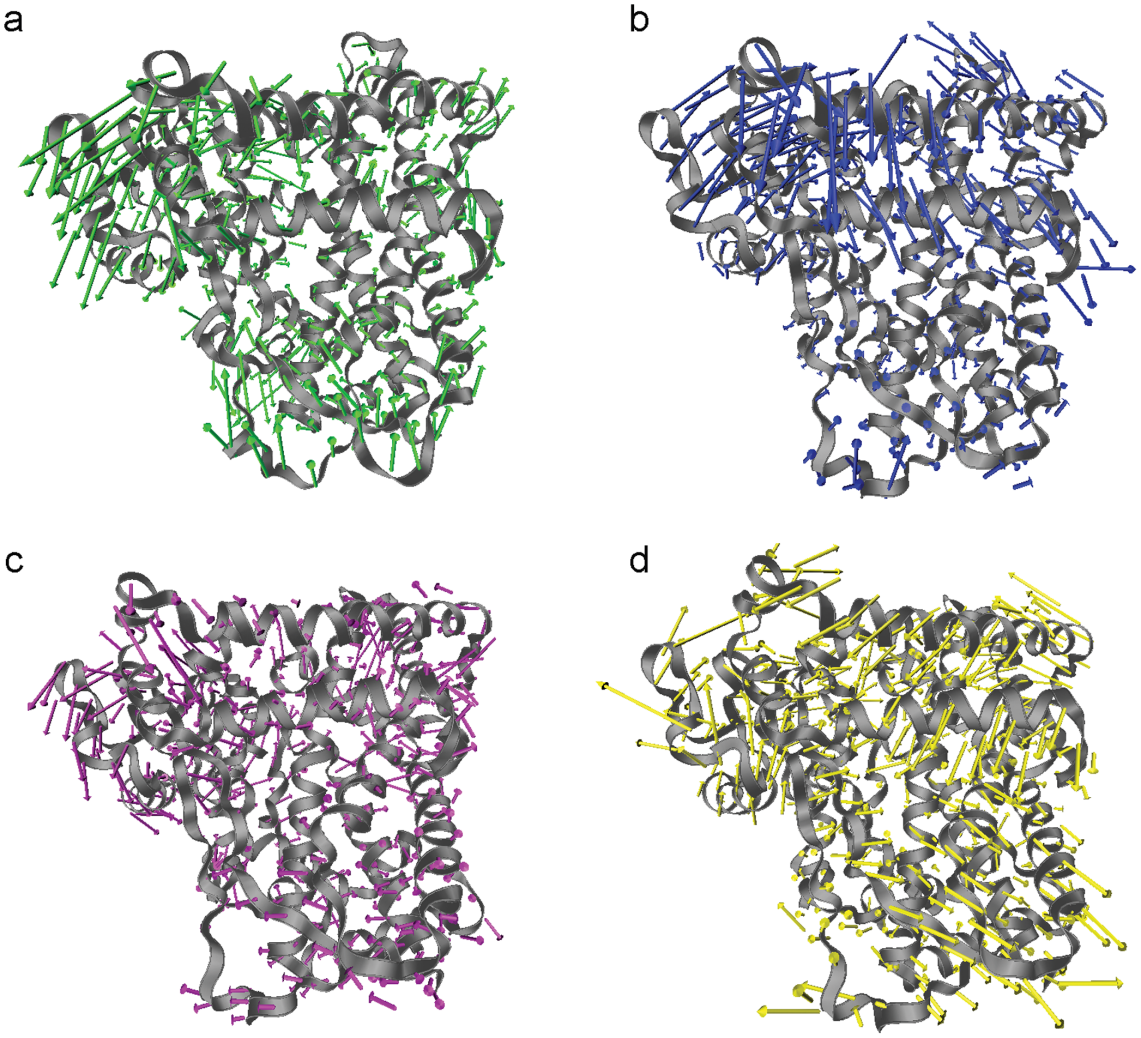
Porcupine plot of the first eigenvector obtained from PCA. Dominant motions of residues in WT (a), R144C (b), I359L (c) and L90P (d) are indicated by arrows on ribbon representation of each variant in different colorings. Magnitude of motions is illustrated by length of arrows.

To determine conformational equilibration during MD simulation, backbone coordinates were fit to the reference structure and measured for RMSD. The simulations converged and plateaued at approximately 12 ns (Figure S1). To determine local collective protein motions during stable simulations, PCA was carried out on the last 4 ns trajectories of wild-type and mutants. For each variant, the first principle component is illustrated in Figure 2. Distinctive motions represented by high magnitude arrows were mostly observed in the flexible regions that have been previously described to influence interaction with substrates [31] such as helices F and G or loop between helices. However, directions of the same flexible region were different across structures. Motions in the core were relatively small in every structure. Therefore, the rigid core was rather stable with low magnitude of movement whereas the flexible region exhibited variable degrees of motion in the same trajectory. Consequently, overall conformations of each snapshot from the last 4 ns simulation were different in the flexible regions. To assess which simulation conformations were most likely to represent a native-like pose with (*S*)-warfarin, the generalized knowledge-based discriminatory function r⋅m⋅r was applied to the same sets of conformations used in PCA.

### Representative Biomolecular Interactions of CYP2C9 Variants

**Figure 3:**
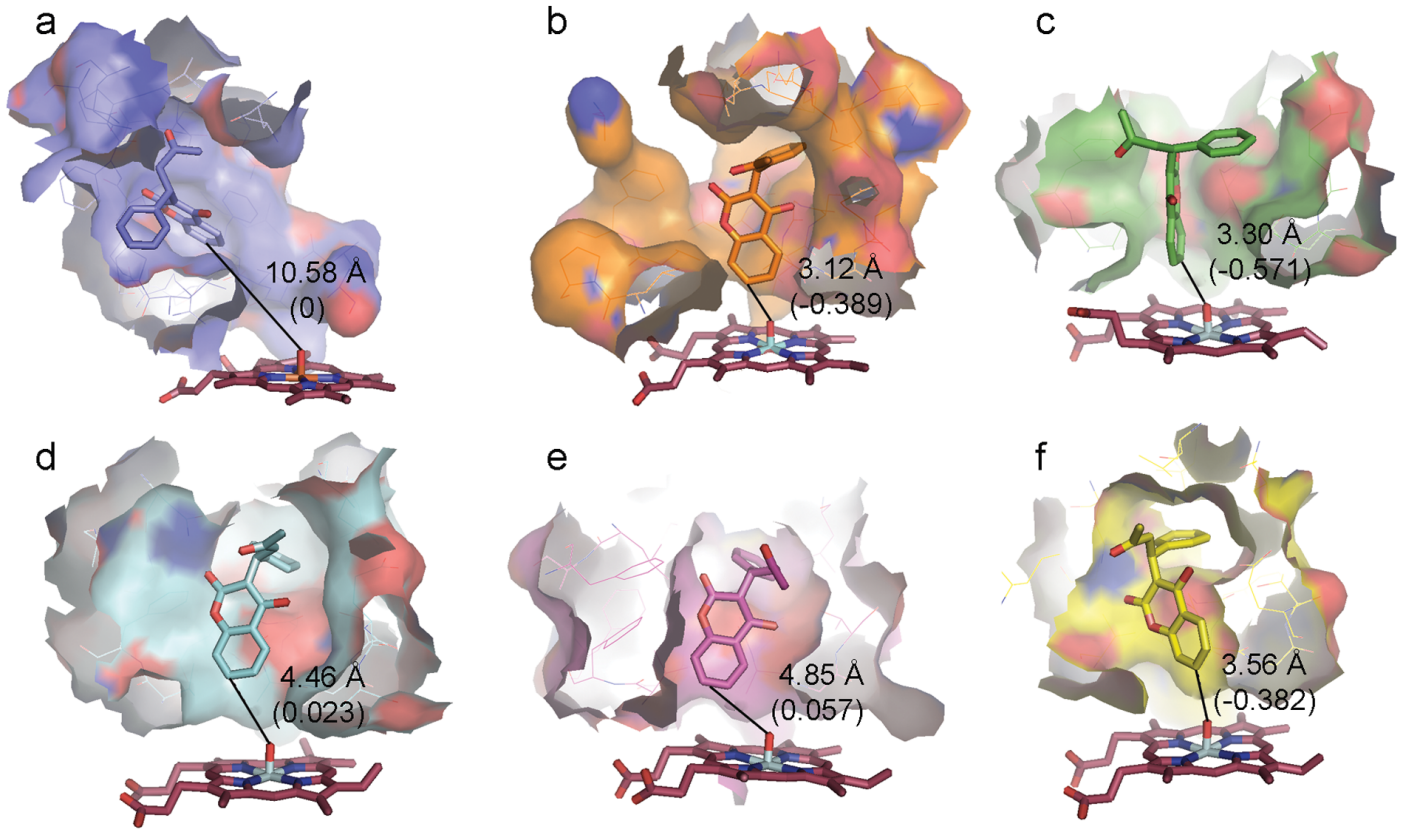
Representative native-like interatomic interactions between (*S*)-warfarin and CYP2C9 variants. The total r⋅m⋅r scores were used to discriminate (*S*)-warfarin-bound conformation from trajectories of each variant. (*S*)-warfarin poses at different distance from heme center in original 1OG5 (a), re-docked conformation in WT (b). Snapshopts from MD simulations were selected for native-like conformation of WT (c), R144C (d), I359L (e), and L90P (f) by means of knowledge-based scores between hydroxylation site C-7 of (S)-warfarin and molecular oxygen (FeO-C7) as shown in parentheses. FeO-C7 distance of each structure is measured in angstrom. Oxyferryl heme is in ruby red stick while (*S*)-warfarin is shown in green (WT), light blue (R144C), magenta (I359L), and yellow (L90P) stick. Oxygen and nitrogen are in red and blue, respectively. Carbon is colored based on CYP2C9 variant.

**Figure 4:**
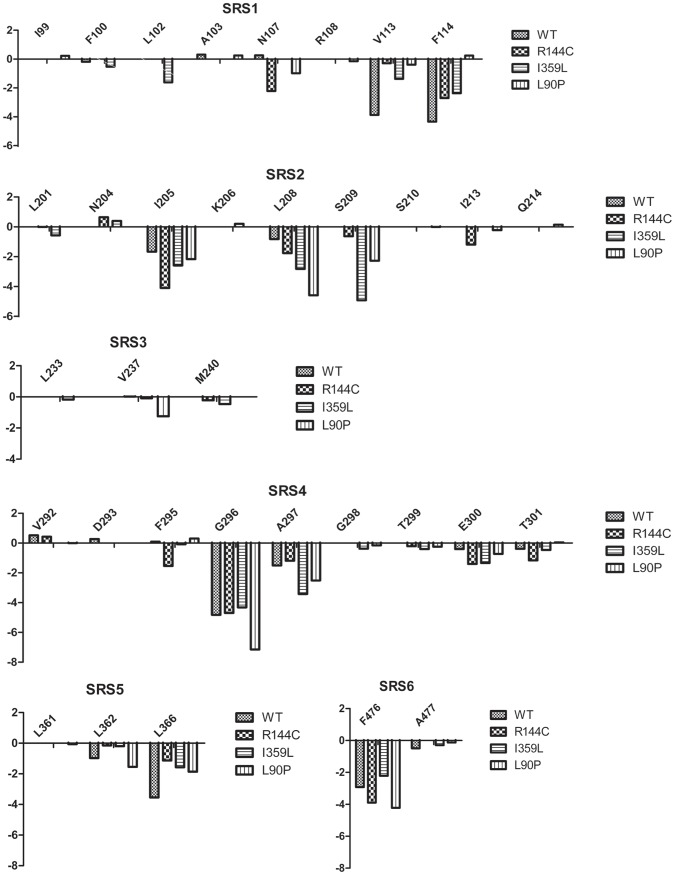
Comparison of r⋅m⋅r scores of residues that are involved in interaction with (*S*)-warfarin. Residues involved in binding with (*S*)-warfarin are scored and classified based on 6 SRSs comprising ligand binding site of CYPs. Favorable interaction is represented by negative score value. Wild-type and mutant residues are designated by different shading of bars.

Interatomic interactions between active-site cavity of the enzyme and (*S*)-warfarin of individual conformation in the final 4 ns of simulations were evaluated by means of r⋅m⋅r scores [33]. For each simulation, conformation with the lowest total r⋅m⋅r score accounting for all interatomic interactions is believed to have the most native-like biomolecular interactions, and the total r⋅m⋅r score of the most native-like conformations obtained from wild-type and mutant simulations are compared in Figure 3 (and Table S1 for detailed data). Since 7-hydroxylated (*S*)-warfarin is the major metabolite from (*S*)-warfarin interacting with the oxyferryl heme of CYP2C9, r⋅m⋅r scores for the interaction between molecular oxygen ligated on iron and attached C-7 are also shown in correlation with the atom pair distance. Upon MD simulations, biomolecular interactions became more favorable (i.e. the r⋅m⋅r scores lowered). The original (*S*)-warfarin-bound crystal structure has r⋅m⋅r score of 0 (no interaction) between molecular oxygen and C7 (FeO-C7), as (*S*)-warfarin is not in close proximity to the heme center (Figure 3a). WT exhibited the best r⋅m⋅r score of FeO-C7 compared to the mutant structures (Figure 3c). L90P showed comparable r⋅m⋅r score of FeO-C7 to that of the docked conformation but a better total r⋅m⋅r score. The FeO-C7 r⋅m⋅r scores of R144C and I359L were less favorable than that of WT, with a longer distance between oxyferryl heme and the hydroxylation site of (*S*)-warfarin (Figure 3d-e). However, the total r⋅m⋅r score was more favorable in R144C and I359L, largely attributable to interactions between (*S*)-warfarin and residues at the top of binding pocket. The r⋅m⋅r scores of individual amino acid residues interacting with (*S*)-warfarin in wild-type and mutant structures are shown in Figure 4. Several residues were found to be involved in the interatomic interactions with (*S*)-warfarin across all variants with different degrees of interactions. The cumulative r⋅m⋅r scores for these residues, averaged across the last 4 ns of the simulations, were −24.728, −25.796, −23.782, and −25.418 in WT, R144C, I359L, and L90P, respectively. Nevertheless, residues in SRS-3 on G-helix of WT were not found to take part in the interatomic interactions with (*S*)-warfarin.

## Discussion

### 1. Global Structural Conformation

To investigate differences in interatomic interactions between (*S*)-warfarin and CYP2C9 variants, a productive orientation of (*S*)-warfarin bound in active-site cavity was predicted by rigid docking and subsequently optimized using MD simulations. Overall structural conformations retained structurally conserved features of CYPs, and low deviation from 1OG5 was observed as RMSD of stable trajectories was less than 2 Å. Dominant motions during simulations were determined by PCA, suggesting that ongoing movements were due to flexible regions while conserved region were stable in the last 4 ns trajectories. Representative complexes from each variant were discriminated by r⋅m⋅r knowledge-based scoring function. The total r⋅m⋅r scores were improved upon optimization of all variant structures, compared to original (*S*)-warfarin-bound crystal 1OG5 and re-docked (*S*)-warfarin in the crystal. Interatomic pair r⋅m⋅r score of FeO-C7 suggests an absence of interaction in 1OG5 in correlation to the large distance between hydroxylation site of (*S*)-warfarin and oxyferryl heme. (*S*)-warfarin was then re-docked in close proximity to heme center and had a negative (favorable) r⋅m⋅r score. During MD simulations, interatomic interactions were optimized, as the total r⋅m⋅r scores are more favorable than the re-docked conformation. Orientations of (*S*)-warfarin in binding pockets of all variants are similar but the distance between oxyferryl heme and C7 on (*S*)-warfarin among variants was different. The FeO-C7 r⋅m⋅r score suggests the strength of heme/substrate interaction of WT to be superior than that of the docked conformation, implying a slightly longer distance obtained during simulation of WT yields a more favorable FeO-C7 interaction. In contrast, (*S*)-warfarin was located much further away from the heme center in R144C and I359L simulations, with positive (unfavorable) FeO-C7 r⋅m⋅r score. The minimum allowed distance between substrate hydroxylation site and heme center was proposed to be 2.9 Å [36]. Larger distance observed in the mutant simulations implies less favorable conformations for metabolic attack, corresponding to the reduction in kinetics activities of R144C and I359L mutants towards (*S*)-warfarin metabolism [9]. FeO-C7 r⋅m⋅r scores also comply with intrinsic clearance (*V*_max_/*K*_m_) for (*S*)-warfarin 7-hydroxylation (WT = 12.2, R144C = 5.0, I359L = 1.3) [9]. Despite less favorable interatomic interaction FeO-C7, the total mutant-r⋅m⋅r scores are largely negative due to contacts with residues on top of the binding pocket. These residues are not observed in wild-type interactions, suggesting favorable (*S*)-warfarin occupancy on top of mutant binding pocket through interatomic interaction with these residues rather than in proximity to heme center as found in WT. In addition, r⋅m⋅r scores between (*S*)-warfarin and key residues F114 and V113 in mutants were worse compared to WT. The r⋅m⋅r score between (*S*)-warfarin and V113 was −0.284, −1.374, and −0.390 in R144C, I359L, and L90P mutants respectively, while the WT score exhibited the most favorable score of −3.872. Moreover, (*S*)-warfarin 7-hydroxylation was shown to be absent in V113L mutation [37], suggesting its important role in metabolism of (*S*)-warfarin in CYP2C9. The r⋅m⋅r score between (*S*)-warfarin and F114 was −2.707, −2.364, and 0.247 in the R144C, I359L, and L90P mutants respectively, while WT score was −4.336. F114 has been shown to dramatically affect enzymatic activities. Activity for 7-hydroxylation of (*S*)-warfarin by F114L was demonstrated to be decreased as *V*_max_ was reduced 4-fold, while *K*_m_ was increased for 13-fold [37]. In addition, a complete loss of diclofenac hydroxylation was observed in F114I mutation [38]. V113 and F114 are essential in enzymatic activity of CYP2C9, and reduction in favorable interactions with V113 and F114 as shown in mutants could affect 7-hydroxylation of (*S*)-warfarin.

### 2. Distal Effects of Single Amino Acid Substitutions

#### 2.1 R144C mutant

**Figure 5:**
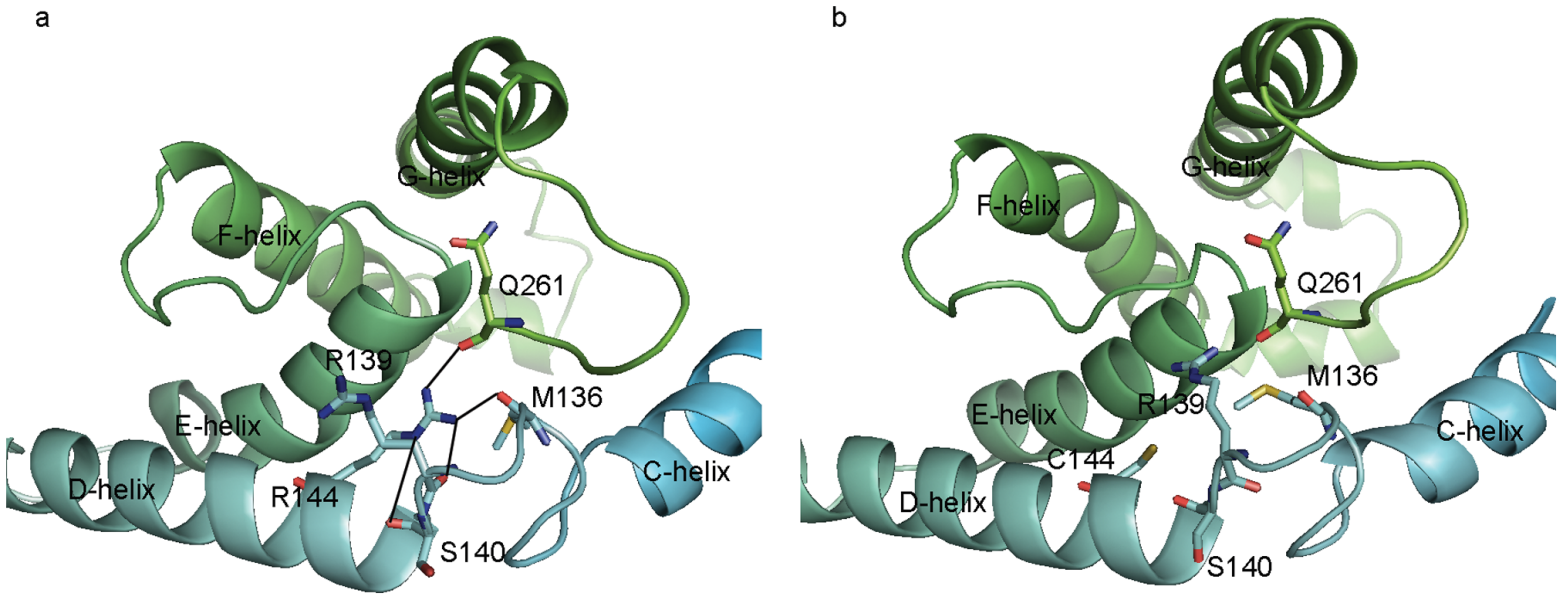
Network of hydrogen bonds formed by R144 in WT. (a) On the surface of the WT structure, helices C, D, and H are held by the network of hydrogen bonds indicated by black lines. The bonds are formed by guanidino group of R144 and carbonyl oxygen on the backbone of M136, R139, S140, and Q261. Such interactions are absent in R144C structure (b) due to a replacement of arginine to cysteine.

Although R144C mutation leads to reduction in enzymatic activity, this residue is located on the surface far from active site of the enzyme. It has been previously suggested that decreased interaction between NADPH-dependent cytochrome P450 reductase (CPR) and R144C could result in reduction in metabolic activity [39]. However, subsequent investigation on metabolism of naproxen by R144C demonstrated comparable CPR interaction as found in WT [40]. In our simulation studies, distance between C7 atom on (*S*)-warfarin and oxyferryl heme was 1.16 Å greater in R144C than that of WT, and r⋅m⋅r score was also higher in R144C, suggesting that even without interaction with CPR, interatomic interaction between R144C and (*S*)-warfarin is less favorable compared to WT. Conformational differences between WT and R144C structure could be ascribed to different formation of hydrogen bond network at the N-terminus of the D-helix. In WT, guanidino moiety of R144 hydrogen bonds to carbonyl groups of M136, R139, S140 on the loop between C-helix and D-helix, and Q261 on the loop between G-helix and H-helix as shown in Figure 5. Such interaction was absent in R144C as hydrogen acceptor is not present. The degree of motion altered in H-helix by disruption of hydrogen bond network could affect orientation of helices F and G that form the roof of binding pocket.

#### 2.2 I359L mutant

CYP2C9*3 represents CYP2C9 variant with I359L mutation that exhibits dramatic reduction in metabolic activity relative to wild-type CYP2C9 [10]. I359L is located close to proposed SRS-5 [19], inferring indirect effect towards metabolic activity. Previous computation study suggested that the space on top of the binding pocket was larger in I359L structure than in WT, such that substrates were not as confined to vicinity of the heme center in this mutant [41]. Replacement of loop between β3.1 and β3.2 was suggested to cause an expansion of the space near F helix [41], that corresponds to PCA result in this study. Furthermore, pair-wise r⋅m⋅r scores revealed a stronger interatomic interaction between S209 on binding pocket roof of I359L and (*S*)-warfarin than that in WT, feasibly to enhance accommodation of (*S*)-warfarin on top of the binding pocket rather than in proximity to the heme center. Owing to this change in (*S*)-warfarin position, its interactions with oxyferryl heme were unfavorable compared to WT as measured by the r⋅m⋅r score. FeO-C7 r⋅m⋅r score of I359L was the least favorable among CYP2C9 variants, in correlation with its FeO-C7 distance. (*S*)-warfarin poses a remarkable distance of 4.85 Å away from oxyferryl heme in this mutant compared to WT distance of 3.30 Å, rendering it to be inferior in catalytic activity to other variants in this study. Previous docking study predicted I359L to be more disruptive mutation than R144C [41]. Regarding to r⋅m⋅r scores and FeO-C7 distance in this study, it could be suggested that I359L might have a lower metabolic activity against (*S*)-warfarin than other variants.

#### 2.3 L90P mutant

CYP2C9*13 is prevalent in Asian populations [15]–[17] and implicated in impaired drug clearance [12]–[14]. However, little is known about an effect of L90P mutation on metabolism of (*S*)-warfarin. A reduction in warfarin dose was observed in a Korean patient with heterozygous CYP2C9*3/*13 [42]. In this study, FeO-C7 r⋅m⋅r score for L90P mutant was slightly lower than that of WT, although FeO-C7 distance was larger than in the WT by 0.26 Å. As in I359L, (*S*)-warfarin has more favorable interactions with residues lining roof of the binding cavity than WT. R108 on B/C loop of L90P particularly interacted strongly with (*S*)-warfarin whereas such interaction was not observed in other variants. Involvement of R108 in binding with (*S*)-warfarin could be attributed to distortion of B/C loop by L90P mutation. Side-chain turn over of residues 106–108 on the same loop was also shown to be responsible for decreased catalytic activity of L90P [43]. Consequently, conformation of B/C loop could be affected by L90P and lead to differences in interactions with drugs.

## Conclusion

Distal effects of single amino acid substitutions in R144C, I359L, and L90P are demonstrated by interatomic interactions towards commonly used anticoagulant (*S*)-warfarin. The mutations are located outside the binding pocket but could result in conformational changes of the pocket, which is greatly flexible and malleable. Consequently, it could be noted that the non-active-site residues could allosterically regulate catalytic activity of the CYP enzymes as previously proposed [44]. Such effects are not limited to CYP2C9 variants demonstrated by this study and elsewhere [41], [43], [45] but also observable in CYP1A2 [44], CYP82E4 [46] and CYP2B4 [47]. Despite a small change in global conformational fold, the non-active-site residues could disrupt residue-residue contacts, leading to destabilization of active-site conformation [44], [47]. As a result, metabolic activity could be impaired through a slight change in active-site topology induced by mutation outside the binding cavity. Using computational approach in this study, distal effects on altered interatomic interactions toward drugs induced by non-active-site mutation of CYP enzymes could be investigated to provide structural insights for characterization of poor drug metabolisms caused by CYP polymorphisms.

## Supporting Information

Figure S1 Backbone atom RMSD. RMSD of backbone coordinates calculated for WT (a), R144C (b), I359L (c), and L90P (d) of 2 replications that are marked separately with different coloring.
(TIF)

Table S1 Measurements of representative (*S*)-warfarin-bound conformation from each variant in comparison to (*S*)-warfarin-bound crystal 1OG5. Abbreviations: RMSD, root mean square deviation; FeO-C7, oxyferry heme and hydroxylation site on C7 of (*S*)-warfarin; r⋅m⋅r, radial distribution function with mean reference state; Docked con., orientation of (*S*)-warfarin obtained from docking. ^1^Distance measured between oxyferryl heme and hydroxylation site of (*S*)-warfarin. ^2^Total r⋅m⋅r score calculated from interatomic interactions between (*S*)-warfarin and the holoenzyme (amino acids and oxyferryl heme). ^3^Pair-wise interatomic interaction between C7 of (*S*)-warfarin and molecular oxygen ligated on heme.
(DOCX)
