# Correction: Distal Effect of Amino Acid Substitutions in CYP2C9 Polymorphic Variants Causes Differences in Interatomic Interactions against *(S)*-Warfarin

**DOI:** 10.1371/annotation/416be1ef-f439-445a-96f8-b1d2f01c6957

**Published:** 2013-09-11

**Authors:** Panida Lertkiatmongkol, Anunchai Assawamakin, George White, Gaurav Chopra, Pornpimol Rongnoparut, Ram Samudrala, Sissades Tongsima

The was an error in the second affiliation, given for authors Panida Lertkiatmongkol and Sissades Tongsima.

This affiliation should read: Genome Institute, National Center for Genetic Engineering and Biotechnology, Pathumtani, Thailand 

