# Supplementary figures and images for: Distal Effect of Amino Acid Substitutions in CYP2C9 Polymorphic Variants Causes Differences in Interatomic Interactions against (S)-Warfarin
Source: PLoS One. 2013 Sep 2;8(9):e74053. doi: 10.1371/journal.pone.0074053 (PMC3759441; doi:10.1371/journal.pone.0074053)

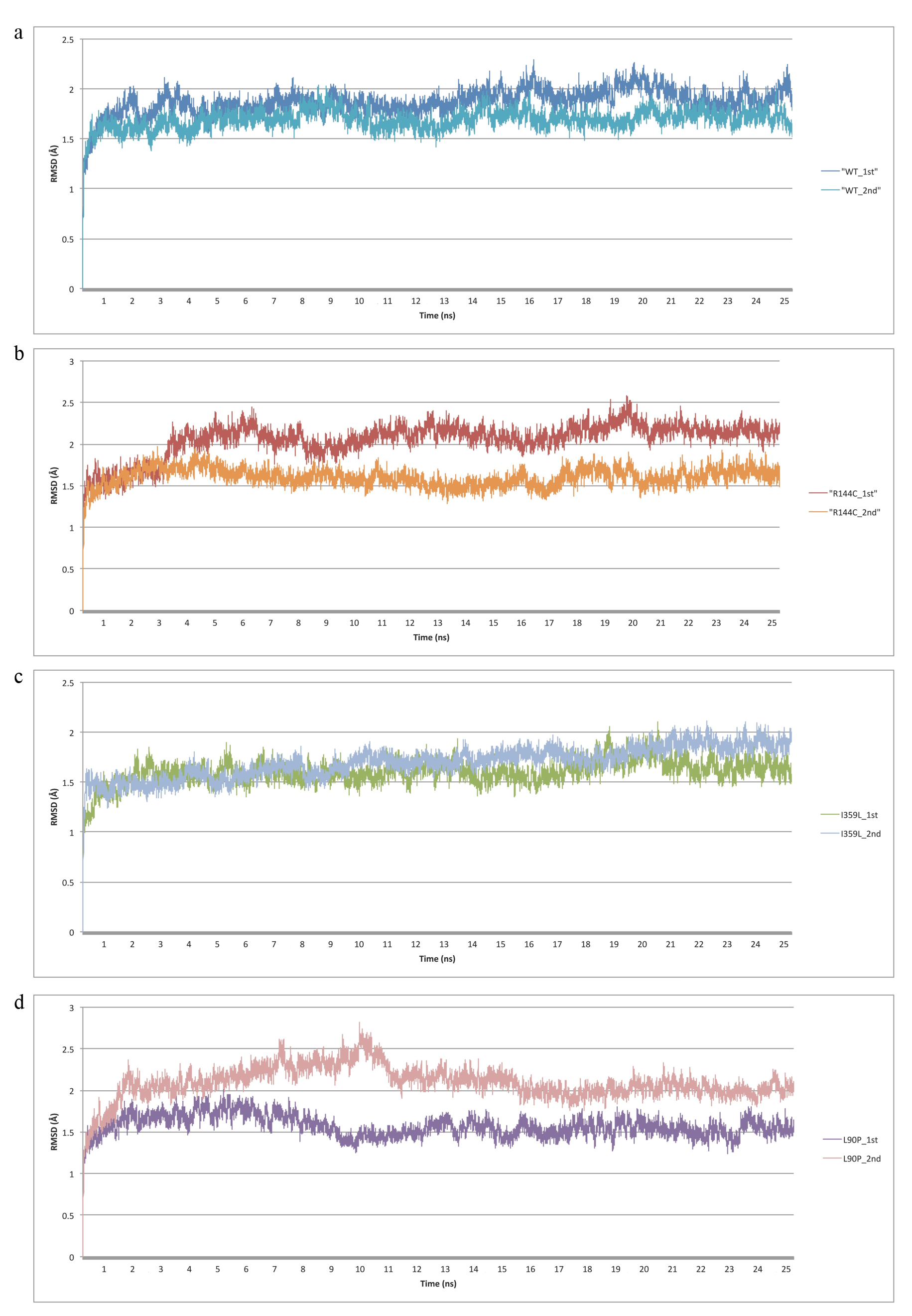

Supplement: Figure S1 — Backbone atom RMSD. RMSD of backbone coordinates calculated for WT (a), R144C (b), I359L (c), and L90P (d) of 2 replications that are marked separately with different coloring. (TIF) [file pone.0074053.s001.tif]
