# Supplementary material for: Distal Effect of Amino Acid Substitutions in CYP2C9 Polymorphic Variants Causes Differences in Interatomic Interactions against (S)-Warfarin
Source: PLoS One. 2013 Sep 2;8(9):e74053. doi: 10.1371/journal.pone.0074053 (PMC3759441; doi:10.1371/journal.pone.0074053)
Supplement: Table S1 — Measurements of representative (S)-warfarin-bound conformation from each variant in comparison to (S)-warfarin-bound crystal 1OG5. Abbreviations: RMSD, root mean square deviation; FeO-C7, oxyferry heme and hydroxylation site on C7 of (S)-warfarin; r⋅m⋅r, radial distribution function with mean reference state; Docked con., orientation of (S)-warfarin obtained from docking. 1Distance measured between oxyferryl heme and hydroxylation site of (S)-warfarin. 2Total r⋅m⋅r score calculated from interatomic interactions between (S)-warfarin and the holoenzyme (amino acids and oxyferryl heme). 3Pair-wise interatomic interaction between C7 of (S)-warfarin and molecular oxygen ligated on heme. (DOCX) [file pone.0074053.s002.docx]

Table S1

| CYP2C9 | | | RMSD from 1OG5 (Å) | FeO-C7 (Å)^1^ | r•m•r score | |
| --- | --- | --- | --- | --- | --- | --- |
|  |  |  |  |  | Total^2^ | FeO-C7^3^ |
| 1OG5  Docked con.  WT  R144C  I359L  L90P | | | 0  0  1.609  1.452  1.274  1.672 | 10.58  3.12  3.30  4.46  4.85  3.56 | 6.258  -3.032  -29.717  -28.009  -37.040  -29.591 | 0  -0.389  -0.571  0.023  0.057  -0.382 |
|  |  |  |  |  |  |  |
